# *CD44* Gene Polymorphisms in Breast Cancer Risk and Prognosis: A Study in North Indian Population

**DOI:** 10.1371/journal.pone.0071073

**Published:** 2013-08-05

**Authors:** Sonam Tulsyan, Gaurav Agarwal, Punita Lal, Sushma Agrawal, Rama Devi Mittal, Balraj Mittal

**Affiliations:** 1 Department of Genetics, Sanjay Gandhi Post Graduate Institute of Medical Sciences, Lucknow, India; 2 Department of Endocrine and Breast Surgery, Sanjay Gandhi Post Graduate Institute of Medical Sciences, Lucknow, India; 3 Department of Radiotherapy, Sanjay Gandhi Post Graduate Institute of Medical Sciences, Lucknow, India; 4 Department of Urology, Sanjay Gandhi Post Graduate Institute of Medical Sciences, Lucknow, India; The University of Texas M. D. Anderson Cancer Center, United States of America

## Abstract

**Background:**

Cell surface biomarker *CD44* plays an important role in breast cancer cell growth, differentiation, invasion, angiogenesis and tumour metastasis. Therefore, we aimed to investigate the role of *CD44* gene polymorphisms in breast cancer risk and prognosis in North Indian population.

**Materials & Methods:**

A total of 258 breast cancer patients and 241 healthy controls were included in the case-control study for risk prediction. According to RECIST, 114 patients who received neo-adjuvant chemotherapy were recruited for the evaluation of breast cancer prognosis. We examined the association of tagging SNP (rs353639) of Hapmap Gujrati Indians in Houston (GIH population) in *CD44* gene along with a significant reported SNP (rs13347) in Chinese population by genotyping using Taqman allelic discrimination assays. Statistical analysis was done using SPSS software, version 17. In-silico analysis for prediction of functional effects was done using F-SNP and FAST-SNP.

**Results:**

No significant association of both the genetic variants of the *CD44* gene polymorphisms was found with breast cancer risk. On performing univariate analysis with clinicopathological characteristics and treatment response, we found significant association of genotype (CT+TT) of rs13347 polymorphism with earlier age of onset (P = 0.029, OR = 0.037). However, significance was lost in multivariate analysis. For rs353639 polymorphism, significant association was seen with clinical tumour size, both at the genotypic (AC+CC) (P = 0.039, OR = 3.02) as well as the allelic (C) (P = 0.042, OR = 2.87) levels. On performing multivariate analysis, increased significance of variant genotype (P = 0.017, OR = 4.29) and allele (P = 0.025, OR = 3.34) of rs353639 was found with clinical tumour size. In-silico analysis using F-SNP, showed altered transcriptional regulation for rs353639 polymorphism.

**Conclusions:**

These findings suggest that *CD44* rs353639 genetic variants may have significant effect in breast cancer prognosis. However, both the polymorphisms- rs13347 and rs353639 had no effect on breast cancer susceptibility.

## Introduction

Breast cancer is the commonest cancer worldwide and second to cervical cancer in women mortality [1]. A large number of environmental and genetic factors are known to play an important role in breast cancer development and prognosis. In recent years, several breast cancer susceptibility genes have been identified with BRCA1 and BRCA2 are major genes related to 15% of hereditary breast cancer cases [2], [3]. Thus, further studies are needed to identify other genes having an impact on breast cancer risk and prognosis which may likely to play a major role in risk prediction.

Breast cancers contain few distinct cells called breast cancer-initiating cells (BCICs), which are characterized by the expression of CIC biomarkers [4]. *CD44* is one such biomarker. *CD44* gene is located on chromosome 11p13 [5]. The encoded protein is a cell surface glycoprotein, involved in a number of biological processes including lymphocyte migration, extravasation, homing, activation and apoptosis [6], [7], [8], [9], [10], [11], [12], [13]. Many studies have also stated its role in tumor metastasis [14], [15]. It is also a receptor for hyaluronic acid. Recent studies have shown *CD44* and its interaction with hyaluronan regulate breast cancer cell proliferation, migration and invasion. In addition genetic variants of *CD44* were found to be associated with breast cancer patient survival, risk prediction and prognosis [16], [17], [18].

SNP rs13347 was previously reported to be significantly associated with breast cancer risk and prognosis in Chinese population [16]. However, in view of limited studies [16], [18], [19], we aimed to determine the association of previously significant reported SNP (rs13347), together with taggerSNP (rs353639) in the *CD44* gene of Hapmap- GIH population with breast cancer risk and prognosis in North Indian population.

## Materials and Methods

### Ethics Statement

The study including the consent process was approved by the ethics committee of Sanjay Gandhi Post Graduate Institute of Medical Sciences (SGPGIMS), Lucknow, India and the authors followed the norms of World’s Association Declaration of Helsinki. Written informed consent was taken from each subject.

### Study Population

The present study consisted of 258 histopathologically confirmed breast cancer patients from north Indian population. Patients were enrolled from the outpatient department (OPD) of Endocrine & Breast Surgery, and Radiotherapy, SGPGIMS, Lucknow, who have completed their treatment as planned between the period from April, 2010 to Oct, 2012. The patients were subjected to detailed demographical, clinical and pathological investigations. Staging of cancer was documented according to the AJCC-TNM classification system [20]. During the same time, age and ethnicity matched 241 healthy controls were recruited from volunteers who came to the hospital for their routine checkups, unrelated to patients and to each other. Selection criteria for controls included no evidence of any personal history of cancer or other malignant conditions.

Out of 258 patients, neo-adjuvant chemotherapy (NACT) was administered to 114 locally advanced or large operable breast cancer (LOBC or LABC) patients. Therefore, case-control study for evaluating breast cancer risk was done in 258 patients while 114 patients were recruited in case only study for evaluation of response to NACT. Pathological response to NACT was recorded according to Response Evaluation Criteria in Solid Tumors (RECIST criteria) [21] and patients were categorized as pathologic complete responders (≥30% tumour regression) and non pathologic responders (<30% tumour regression).

### TagSNPs Selection

TagSNPs were selected from the Haploview software 4.2 (Mark Daly’s lab of Broad Institute, Cambridge, MA, Britain) [22], based on the GIH population data of HapMap (HapMap Data Rel 27 PhaseII +III, Feb 09, on NCBI B36 assembly, dbSNP b126). TagSNPs that captured all the known common SNPs (with minor allele frequencies of >0.1) in the *CD44* gene, with a pairwise correlation r^2^>0.8 were selected.

**Figure 1:**
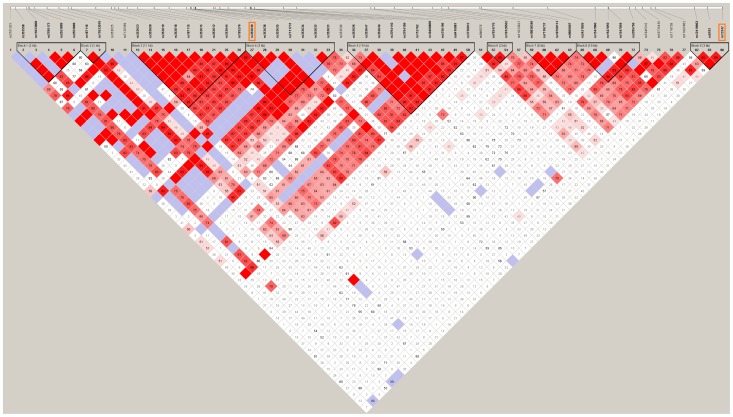
Linkage disequilibrium (LD) plot of *CD44* gene in Hapmap- GIH population.

TaggerSNP rs 353639 was found to represent the known SNPs in the haplotype blocks 3 and 4 in the *CD44* gene of GIH population (Figure S1). It was also found to represent the haplotype block 5 in Caucasian (CEU in Hapmap) population (Figure S1). Previously significantly reported SNP (rs13347) in Chinese population also represents the haplotype block 9 (Figure 1).

### Genotyping

Genomic DNA was extracted from the venous blood using the standard salting out method [23]. The quality and quantity of DNA was checked by using Nanodrop spectrophotometer (Thermo Fisher Scientific/Nanodrop Products, Wilmington, Delaware, USA). Genotyping of both the SNPs: rs13347 and rs353639 was carried out using Taqman allelic discrimination assay. Primers and probes were supplied as pre-designed assays by Applied Biosystems (Foster City, CA, USA). Genotyping was performed on an ABI 7500 Real Time PCR system using 96-well plates. All plates included negative controls (wells containing no DNA). ABI PRISM 7500 sequence detection software was used for amplification detection.

### Statistical Analysis

Effective sample sizes for case-control study was calculated by Quanto 1.1 ver. software and the power was set at 80% [24]. Descriptive statistics of patients and controls were presented as the mean and standard deviations (SDs) for continuous measures, while frequencies and percentages were used for categorical measures. The null hypothesis that the Hardy–Weinberg equilibrium holds was tested using a chi-squared test for deviation from Hardy–Weinberg equilibrium. The relationship between genetic variants and clinicopathological features along with breast cancer treatment response was examined using univariate analysis through Fisher’s exact test. Similarly, multivariate analysis was also performed by using binary logistic regression. Clinicopathological features included in the analysis were age, tumor size, HER2 status, hormone receptor status, histology, grade and nodal status. All statistical analysis was done using SPSS statistical analysis software, version 17.0 (SPSS, Chicago, IL, USA). Association was expressed as odds ratios (OR) with 95% confidence intervals (CI). The association was considered to be significant when the *P*-value was <0.05.

### In Silico Analysis

The possible functional effects were determined in *CD44* gene by online web servers FASTSNP (http://fastsnp.ibms.sinica.edu.tw) and F-SNP (http://compbio.cs.queensu.ca/F-SNP/) [25], [26].

## Results

### Population Characteristics

The detailed demographic, clinical and pathological characteristics of study subjects were illustrated in Table 1.

**Table 1:** Characteristics of the study population.

Characteristics		258 Cases	241 Controls
		N (%)	N (%)
Age (mean±SD)		49.47±11.082	47.42±10.385
Menstrual status	Pre	98 (38.0)	127 (52.7)
	Post	160 (62.0)	114 (47.3)
Histology	Ductal	242 (93.8)	
	Lobular	4 (1.6)	
	Mixed	8 (3.1)	
	Missing data	4 (1.6)	
Grade (G)	Negative	11 (4.3)	
	G2	144 (55.8)	
	G3	96 (37.2)	
	Missing data	7 (2.7)	
Clinical tumour size (cm)	0	3 (1.2)	
	<2	22 (8.5)	
	2.1–5.0	119 (46.1)	
	>5.0	94 (36.4)	
	Missing data	20 (7.8)	
Clinical lymph node	0	87 (33.7)	
	<3	102 (39.5)	
	4–9	48 (18.6)	
	>10	10 (3.9)	
	Missing data	11 (4.3)	
Metastasis	M0	240 (93.8)	
	M1	16 (6.2)	
Hormone Receptor	Positive	117 (45.3)	
	Negative	136 (52.7)	
	Missing data	5 (2.0)	
Her 2 neu expression	Positive	91 (35.3)	
	Negative	150 (58.1)	
	Missing data	17 (6.6)	
Treatment given	NACT	114 (44.2)	
	ACT	130 (50.4)	
	Missing data	14 (5.4)	

### *CD44* gene Polymorphisms with Breast Cancer Risk

The observed genotype frequencies of the two polymorphisms studied in healthy controls were in accordance with Hardy-Weinberg equilibrium.

Table 2 shows the risk of breast cancer in context with each of the SNPs studied in *CD44* gene. No significant differences were observed in the frequency distribution of rs13347 and rs353639 polymorphisms between breast cancer patients and healthy controls, both at the genotypic and allelic levels. Further analyzing our study subjects on stratification based on menstrual status, we did not find significant correlations of the genotypes as well as alleles of both the polymorphisms with breast cancer risk.

**Table 2:** Association of rs13347 and rs353639 polymorphisms with breast cancer risk.

Genotypes/Alleles	Overall				Premenopausal				Postmenopausal			
	Cases(%)	Controls(%)	#OR(95% CI)	P value	Cases	Controls	#OR(95% CI)	P value	Cases	Controls	#OR(95% CI)	P value
rs13347												
CC	191 (74.0)	178 (73.9)	1 (Reference)	–	71 (72.4)	96(75.6)	1 (Reference)	–	120 (75.0)	82 (71.9)	1 (Reference)	–
CT	60 (23.3)	57 (23.7)	1.00 (0.65–1.53)	0.983	24 (24.5)	29 (22.8)	1.15 (0.61–2.15)	0.659	36 (22.5)	28 (24.6)	0.87 (0.48–1.56)	0.645
TT	7 (2.7)	6 (2.5)	1.16 (0.37–3.60)	0.792	3 (3.1)	2 (1.6)	2.22 (0.35–13.80)	0.392	4 (2.5)	4 (3.5)	0.74 (0.17–3.06)	0.678
CT+TT	67 (26.0)	63 (26.1)	1.02 (0.67–1.53)	0.926	27 (27.6)	31(24.4)	1.21 (0.66–2.22)	0.527	40 (25.0)	32 (28.1)	0.856 (0.49–1.49)	0.582
C	442 (85.7)	413 (85.7)	1 (Reference)	–	166 (84.7)	221 (87.0)	1 (Reference)	–	276 (86.2)	192 (84.2)	1 (Reference)	–
T	74 (14.3)	69 (14.3)	1.03 (0.71–1.48)	0.867	30 (15.3)	33(13.0)	1.25 (0.73–2.13)	0.416	44 (13.8)	36 (15.8)	0.85 (0.52–1.39)	0.540
rs353639												
AA	158 (61.2)	150 (61.2)	1 (Reference)	–	60 (61.2)	76 (59.8)	1 (Reference)	–	98 (61.2)	74 (64.9)	1 (Reference)	–
AC	89 (34.5)	89 (34.5)	1.16 (0.78–1.70)	0.451	35 (35.7)	45 (35.4)	0.99 (0.56–1.73)	0.978	54 (33.8)	33 (28.9)	1.36 (0.79–2.34)	0.263
CC	11 (4.3)	13 (5.4)	0.75 (0.31–1.77)	0.514	3 (3.1)	6 (4.7)	0.65 (0.15–2.73)	0.560	8 (5.0)	7 (6.1)	0.78 (0.26–2.31)	0.657
AC+CC	100 (38.8)	91 (37.8)	1.09 (0.75–1.59)	0.618	38 (38.8)	51 (40.2)	0.95 (0.55–1.63)	0.862	62 (38.8)	40 (35.1)	1.25 (0.75–2.08)	0.387
A	405 (78.5)	378 (78.4)	1 (Reference)	–	155 (79.1)	197 (77.6)	1 (Reference)	–	250 (78.1)	181 (79.4)	1 (Reference)	–
C	111 (21.5)	104 (21.6)	1.02 (0.75–1.39)	0.897	41 (20.9)	57 (22.4)	0.92 (0.58–1.45)	0.731	70 (21.9)	47 (20.6)	1.10 (0.72–1.69)	0.636

OR = Odd’s Ratio; CI = Confidence Interval.

#OR adjusted by age.

### *CD44* gene polymorphisms with Breast Cancer Prognosis

Univariate analysis by Fisher’s exact test and Multivariate by logistic regression was employed to correlate the genotypes of the two *CD44* polymorphisms with the clinicopathological features and breast cancer pathologic response to NACT.

In univariate analysis, we found significant correlations of genotype (CT+TT) of rs13347 polymorphism with earlier age of onset (P = 0.029, OR = 0.037) (Table 3). However, we could not find any association at the allelic level (Table 3). The data also revealed significant association of rs353639 polymorphism with clinical tumour size, both at the genotypic (AC+CC) (P = 0.039, OR = 3.02) as well as the allelic (P = 0.042, OR = 2.87) levels (Table 4).

**Table 3:** Univariate analysis of the association of rs13347 polymorphism with breast cancer clinicopathological features and treatment response.

		rs13347							
		CC	CT+TT	OR (95% CI)	*P value	C	T	OR (95% CI)	*P value
Age (years)	≤45	27 (62.8)	16 (37.2)	1 (Reference)	–	70 (81.4)	16 (18.6)	1 (Reference)	–
	>45	58 (81.7)	13 (18.3)	0.37 (0.16–0.89)	**0.029**	127 (89.4)	15 (10.6)	0.51 (0.24–1.10)	0.110
Clinical Tumour size (cm)	<5	25 (86.2)	4 (13.8)	1 (Reference)	–	54 (93.1)	4 (6.9)	1 (Reference)	–
	>5	53 (70.7)	22 (29.3)	2.59 (0.80–8.33)	0.132	126 (84.)	24 (16.0)	2.57 (0.85–7.76)	0.112
Clinical lymph node	Negative	7 (70.0)	3 (30.0)	1 (Reference)	–	17 (85.0)	3 (15.0)	1 (Reference)	–
	Positive	74 (74.0)	26 (26.0)	0.82 (0.19–3.40)	0.722	172 (86.0)	28 (14.0)	0.92 (0.25–3.35)	1.000
Hormone Receptor	Negative	39 (75.0)	13 (25.0)	1 (Reference)	–	91 (87.5)	13 (12.5)	1 (Reference)	–
	Positive	46 (75.4)	15 (24.6)	0.97 (0.41–2.30)	1.000	105 (86.1)	17 (13.9)	1.13 (0.52–2.45)	0.845
Her 2 neu expression	Negative	50 (75.8)	16 (24.2)	1 (Reference)	–	115 (87.1)	17 (12.9)	1 (Reference)	–
	Positive	32 (74.4)	11 (25.6)	1.07 (0.44–2.60)	1.000	74 (86.0)	12 (14.0)	1.09 (0.49–2.42)	0.840
Grade	G1+G2	49 (76.6)	15 (23.4)	1 (Reference)	–	112 (87.5)	16 (12.5)	1 (Reference)	–
	G3	36 (72.0)	14 (28.0)	1.27 (0.54–2.96)	0.666	85 (85.0)	15 (15.0)	1.23 (0.57–2.63)	0.698
Treatment Response	pCR	52 (76.5)	16 (23.5)	1 (Reference)	–	119 (87.5)	17 (12.5)	1 (Reference)	–
	No pCR	33 (71.7)	13 (28.3)	1.28 (0.54–3.00)	0.662	78 (84.8)	14 (15.2)	1.25 (0.58–2.69)	0.561

OR = Odd’s Ratio; CI = Confidence Interval; Significant P value <0.05 is given in bold.

pCR = pathologic complete response.

* Fisher’s exact test.

**Table 4:** Univariate analysis of the association of rs353639 polymorphism with breast cancer clinicopathological features and treatment response.

		rs353639							
		AA	AC+CC	OR (95% CI)	*P value	A	C	OR (95% CI)	*P value
Age (years)	<45	31 (72.1)	12 (27.9)	1 (Reference)	–	72 (83.7)	14 (16.3)	1 (Reference)	–
	>45	44 (62.0)	27 (38.0)	1.58 (0.69–3.60)	0.312	112 (78.9)	30 (21.1)	1.37 (0.68–2.77)	0.393
Clinical Tumour size (cm)	<5	24 (82.8)	5 (17.2)	1 (Reference)	–	53 (91.4)	5 (8.6)	1 (Reference)	–
	>5	46 (61.3)	29 (38.7)	3.02 (1.03–8.82)	**0.039**	118 (78.7)	32 (21.3)	2.87 (1.06–7.78)	**0.042**
Clinical lymph node	Negative	5 (50.0)	5 (50.0)	1 (Reference)	–	15 (75.0)	5 (25.0)	1 (Reference)	–
	Positive	67 (67.0)	33 (33.0)	0.49 (0.13–1.82)	0.310	162 (81.0)	38 (19.0)	0.70 (0.24–2.05)	0.555
Hormone Receptor	Negative	34 (65.4)	18 (34.6)	1 (Reference)	–	83 (79.8)	21 (20.2)	1 (Reference)	–
	Positive	40 (65.6)	21 (34.4)	0.99 (0.65–1.51)	1.000	99 (81.1)	23 (18.9)	0.91 (0.47–1.77)	0.867
Her 2 neu expression	Negative	44 (66.7)	22 (33.3)	1 (Reference)	–	108 (81.8)	24 (18.2)	1 (Reference)	–
	Positive	28 (65.1)	15 (34.9)	1.07 (0.47–2.40)	1.000	69 (80.2)	17 (19.8)	1.10 (0.55–2.21)	0.860
Grade	G1+G2	41 (64.1)	23 (35.9)	1 (Reference)	–	102 (79.7)	26 (20.3)	1 (Reference)	–
	G3	34 (68.0)	16 (32.0)	0.83 (0.38–1.83)	0.695	82 (82.0)	18 (18.0)	0.86 (0.44–1.67)	0.736
Treatment Response	pCR	46 (67.6)	22 (32.4)	1 (Reference)	–	111 (81.6)	25 (18.4)	1 (Reference)	–
	No pCR	29 (63.0)	17 (37.0)	1.22 (0.55–2.68)	0.689	73 (79.3)	19 (20.7)	1.15 (0.59–2.24)	0.733

OR = Odd’s Ratio; CI = Confidence Interval; Significant P values <0.05 are given in bold.

pCR = pathologic complete response.

* Fisher’s exact test.

Next, we performed a multivariate analysis using a logistic regression to evaluate the correlations of both the *CD44* gene polymorphisms with the clinicopathological features and breast cancer treatment response to NACT. In rs13347 polymorphism, we observed that significant association of genotypes with earlier age of onset was lost on applying multivariate analysis (Table 5). No significant association of the alleles with any of the clinicopathological features as well as treatment response was seen (Table 5). On analyzing the data in rs353639 polymorphism with logistic regression, we found increased significance of both the genotype (P = 0.017, OR = 4.29) as well as allele (P = 0.025, OR = 3.34) with clinical tumour size when compared with the results of univariate analysis (Table 6). However, no significant association of both the polymorphisms was seen with treatment response to NACT.

**Table 5:** Multivariate analysis of the association of rs13347polymorphism with breast cancer clinicopathological features and treatment response.

	rs13347			
	CT+TT vs. CC		T vs. C	
	OR (95% CI)	P value	OR (95% CI)	P value
Age (>45 years vs. <45years)	0.70 (0.26–1.84)	0.473	0.91 (0.38–2.16)	0.832
Clinical Tumour size (>5cm vs. <5cm)	2.62 (0.76–9.06)	0.126	2.74 (0.85–8.79)	0.089
Clinical lymph node (positive vs. negative)	0.56 (0.12–2.610	0.466	0.63 (0.16–2.51)	0.520
Hormone Receptor (positive vs. negative)	1.05 (0.37–2.91)	0.924	1.30 (0.52–3.28)	0.569
Her 2 neu expression (positive vs. negative)	1.09 (0.41–2.91)	0.853	1.13 (0.47–2.70)	0.776
Grade (G2+G3 vs. G1)	1.50 (0.54–4.16)	0.430	1.56 (0.63–3.86)	0.334
Treatment Response (No pCR vs. pCR)	1.55 (0.58–4.15)	0.375	1.54 (0.64–3.68)	0.325

OR = Odd’s Ratio; CI = Confidence Interval.

pCR = pathologic complete response.

**Table 6:** Multivariate analysis of the association of rs353639 polymorphism with breast cancer clinicopathological features and treatment response.

	rs353639			
	AC+CC vs. AA		C vs. A	
	OR (95% CI)	P value	OR (95% CI)	P value
Age (>45 years vs. <45years)	2.30 (0.87–6.06)	0.091	1.74 (0.76–3.98)	0.185
Clinical Tumour size (>5cm vs. <5cm)	4.29 (1.30–14.11)	**0.017**	3.34 (1.16–9.59)	**0.025**
Clinical lymph node (positive vs. negative)	0.29 (0.06–1.31)	0.109	0.48 (0.14–1.58)	0.229
Hormone Receptor (positive vs. negative)	1.20 (0.44–3.21)	0.713	1.08 (0.47–2.510	0.844
Her 2 neu expression (positive vs. negative)	1.26 (0.50–3.20)	0.616	1.21 (0.54–2.66)	0.635
Grade (G2+G3 vs. G1)	1.09 (0.40–2.92)	0.863	0.92 (0.39–2.18)	0.861
Treatment Response (No pCR vs. pCR)	1.88 (0.73–4.81)	0.188	1.63 (0.74–3.58)	0.220

OR = Odd’s Ratio; CI = Confidence Interval; Significant P values <0.05 are given in bold.

pCR = pathologic complete response.

### In Silico Analysis of *CD44* gene Polymorphisms

SNPs- rs13347 and rs353639 selected for the present study are located in 3′ UTR and intron region (non-coding sequences) of the *CD44* gene. Therefore, it was possible that this SNP may effect the transcription of the gene. In-silico analysis using F-SNP showed change in transcriptional regulation for both the selected SNPs (Table 7).

**Table 7:** In Silico analysis of *CD44* gene polymorphisms by F SNP and FAST SNP.

Polymorphisms	F SNP				FAST SNP	
	Functional Category	Prediction Tool	Prediction Result	FS Score	Possible Functional Effects	Risk
rs13347	Transcriptional regulation	TFSearch	changed	0.176	Downstream with no known function	Unknown-Unknown (0–0)
		GoldenPath	not exist			
rs353639	Transcriptional regulation	TFSearch	changed	0.176	Intronic with no known function	Unknown-Unknown (0–0)
		GoldenPath	not exist			

## Discussion

*CD44* is a transmembrane glycoprotein involved in many functions such as cell proliferation, angiogenesis, invasion and metastasis [27]. *CD44* gene is composed of 20 exons [5] in two groups. One group consists of exons 1–5 and 16–20, which are expressed together whereas the other group has exons from 6–15. These 10 exons are alternatively spliced and can be included within the group one exons- 5 and 16. Multi-functional nature of the *CD44* depends on the binding of its ligand- hyaluronic acid [28]. There are two binding domains for hyaluronan- exon 2 and exon 5 [29]. Interaction of *CD44* with hyaluronan is involved in the regulation of breast cancer through cell-cell adhesion and inhibited invasion [30]. However, altered binding of hyaluronan to *CD44* can activate cell growth, survival, invasion and metastasis in breast cancer [31], [32] as well as in other cancers [33], [34]. On the basis of above studies, we can state that *CD44* has a significant role in cancer development and prognosis. Therefore, the present study was carried out to evaluate the role of *CD44* gene polymorphisms in north Indian breast cancer patients.

Two polymorphisms within the *CD44* gene were studied to investigate the association of genetic variants with breast cancer risk prediction and prognosis. TaggerSNP approach was used to select the SNP (rs353639) that represents all known SNPs in the *CD44* gene of GIH population. The previously significant reported SNP (rs13347) in Chinese breast cancer patients was also selected to replicate the results in our population. *CD44* gene polymorphisms have not been widely studied with only few reports in breast cancer worldwide [16], [17], [18], [19]. However till date, there is no report on influence of rs353639 polymorphism except a Genome-wide association report for subclinical atherosclerosis in the NHLBI's Framingham Heart Study [35].

In this study, we sought to determine genetic variants of *CD44* that may confer individual’s risk to breast cancer in 258 patients and 131 healthy controls. However, we did not observe any significant differences in the frequency distribution of the genetic variants *CD44* rs13347 between breast cancer patients and healthy controls. After stratification of our subjects on the basis of menstrual status also, no significant association was found. Our results are not in agreement with the single reported study on rs13347 polymorphism with susceptibility to breast cancer [16]. This study by Jiang et al., evaluated the variations in rs13347 polymorphism in 1,853 breast cancer patients and 1,992 healthy control subjects of Chinese population and variant genotype (CT+TT) conferred 1.72 times increased susceptibility to breast cancer. They also performed reporter assay to validate their findings and found variant genotype (CT+TT) carriers to have more *CD44* expression than wild type (CC) carriers. Reasons for variations in results can be due to difference in ethnicity and it is possible that another linked SNP of *CD44* may be conferring risk in our population.

Therefore, one taggerSNP (rs353639) in the *CD44* gene of GIH population was selected to evaluate the effect on breast cancer risk. However, we still found no significant association of rs353639 polymorphism with susceptibility to breast cancer. We also did not find any association on sub-group analysis based on menstrual status. However, no study on the role of rs353639 polymorphism in cancer risk has been reported. Therefore, reasons for discrepancy in the effect of *CD44* polymorphisms on breast cancer susceptibility may be because these polymorphisms have indirect role on breast cancer susceptibility. And their effects may possibly be mediated through linkage to some other key functional polymorphisms, especially in exon 2 and 5 regions of hyaluronan binding of CD44.

A study by Zhou et al., reported a unique SNP *CD44* Ex2+14 A>G in the intron 1 region and found that patients with variant genotype had breast cancer at earlier ages, larger tumor burden, more regional lymph node metastasis and higher cancer recurrence [19]. Another study published by same author in 2012, identified 4 SNPs in exon 2 through sequencing and found significant association of the *CD44* polymorphisms in exon 2 coding sequence with higher probability and higher cumulative risk for breast cancer [36]. A recent study also showed significant increase in the *CD44* expression in breast cancer when compared to normal breast epithelium [37]. Thus, based on conflicting findings, there is a need to replicate the above findings in larger sample size of different ethnicity to come to a definitive conclusion.

We also investigated the effect of genetic variants of *CD44* gene polymorphisms with clinicopathological features and pathologic response to NACT in a cohort of 114 patients. For rs13347 polymorphism, significant association of genotype (CT+TT) with earlier age of onset was found in univariate analysis but was lost on applying multivariate logistic regression. It therefore highlighted that genetic variants do not alone play a role in breast cancer development and prognosis. It is also necessary to evaluate the role of confounding factors like age, clinical stage, pathological lymph node, grade, hormone and Her 2 neu receptor along with these known variants. Similarly, no significant difference of genotype and allele of this polymorphism was seen with pathologic response to NACT. On the contrary, Yao et al, 2009 observed higher expression of *CD44* before chemotherapy in drug resistant cell lines than drug sensitive ones [38]. A study by Zhou et al., reported variant genotype of *CD44* rs4756195 polymorphism to be associated with response to anthracyclines based chemotherapy in patients with breast cancer [18].

For GIH tagger SNP rs353639, we found variant genotype (AC+CC) and allele (C) to be significantly associated with increased clinical tumour size. These results were consistent even after multivariate analysis. Our findings are in agreement with the concept that genetic variations in *CD44* gene may possibly effect the altered binding of its ligand- hyaluronan which leads to increased breast cancer cell growth and differentiation. The importance of this result was strengthened by performing bioinformatic analysis for prediction of functional effects for *CD44* rs353639 polymorphism. Possible functional mechanisms include altered *CD44* expression by differential binding of transcription factors.

Similar to rs13347 polymorphism, no association of genetic variants of *CD44* rs353639 polymorphism was seen with pathologic response to NACT. Therefore, influence of *CD44* gene polymorphisms in breast cancer treatment response to NACT is still not established.

### Conclusions

Our study indicated that both the polymorphisms in *CD44* gene might not have any effect on breast cancer risk prediction in north Indian population. But these polymorphisms have definitely some implications in breast cancer prognosis. To our best knowledge, present study is the first to report a taggerSNP based selection of *CD44* gene polymorphisms with breast cancer risk and prognosis in North Indian subjects. However, study may require confirmation in larger population based cohorts. Furthermore, our findings need to be validated in breast cancer patients of different ethnicities with a gene expression functional assay.

## Supporting Information

Figure S1 **Linkage disequilibrium (LD) plot of** ***CD44*** **gene in Hapmap- CEU population.**
(TIF)

## References

[1] Hortobagyi GN, de la Garza Salazar J, Pritchard K, Amadori D, Haidinger R, et al (2005) The global breast cancer burden: variations in epidemiology and survival. Clin Breast Cancer 6: 391–401. 16381622 10.3816/cbc.2005.n.043

[2] Peto J, Collins N, Barfoot R, Seal S, Warren W, et al (1999) Prevalence of BRCA1 and BRCA2 gene mutations in patients with early-onset breast cancer. J Natl Cancer Inst 91: 943–949. 10359546 10.1093/jnci/91.11.943

[3] Prevalence and penetrance of BRCA1 and BRCA2 mutations in a population-based series of breast cancer cases. Anglian Breast Cancer Study Group. Br J Cancer 83: 1301–1308. 10.1054/bjoc.2000.1407 PMC2408797 11044354

[4] Lobo NA, Shimono Y, Qian D, Clarke MF (2007) The biology of cancer stem cells. Annu Rev Cell Dev Biol 23: 675–699. 17645413 10.1146/annurev.cellbio.22.010305.104154

[5] Goodfellow PN, Banting G, Wiles MV, Tunnacliffe A, Parkar M, et al (1982) The gene, MIC4, which controls expression of the antigen defined by monoclonal antibody F10.44.2, is on human chromosome 11. Eur J Immunol 12: 659–663. 7140811 10.1002/eji.1830120807

[6] Bourguignon LY, Zhu D, Zhu H (1998) CD44 isoform-cytoskeleton interaction in oncogenic signaling and tumor progression. Front Biosci 3: d637–649. 9634539 10.2741/a308

[7] Seth A, Gote L, Nagarkatti M, Nagarkatti PS (1991) T-cell-receptor-independent activation of cytolytic activity of cytotoxic T lymphocytes mediated through CD44 and gp90MEL-14. Proc Natl Acad Sci U S A 88: 7877–7881. 1881921 10.1073/pnas.88.17.7877 PMC52407

[8] Rafi A, Nagarkatti M, Nagarkatti PS (1997) Hyaluronate-CD44 interactions can induce murine B-cell activation. Blood 89: 2901–2908. 9108410

[9] Chen D, McKallip RJ, Zeytun A, Do Y, Lombard C, et al (2001) CD44-deficient mice exhibit enhanced hepatitis after concanavalin A injection: evidence for involvement of CD44 in activation-induced cell death. J Immunol 166: 5889–5897. 11342603 10.4049/jimmunol.166.10.5889

[10] Al-Hajj M, Wicha MS, Benito-Hernandez A, Morrison SJ, Clarke MF (2003) Prospective identification of tumorigenic breast cancer cells. Proc Natl Acad Sci U S A 100: 3983–3988. 12629218 10.1073/pnas.0530291100 PMC153034

[11] McKallip RJ, Fisher M, Do Y, Szakal AK, Gunthert U, et al (2003) Targeted deletion of CD44v7 exon leads to decreased endothelial cell injury but not tumor cell killing mediated by interleukin-2-activated cytolytic lymphocytes. J Biol Chem 278: 43818–43830. 12904302 10.1074/jbc.M304467200

[12] McKallip RJ, Fisher M, Gunthert U, Szakal AK, Nagarkatti PS, et al (2005) Role of CD44 and its v7 isoform in staphylococcal enterotoxin B-induced toxic shock: CD44 deficiency on hepatic mononuclear cells leads to reduced activation-induced apoptosis that results in increased liver damage. Infect Immun 73: 50–61. 15618140 10.1128/IAI.73.1.50-61.2005 PMC538933

[13] Sales KM, Winslet MC, Seifalian AM (2007) Stem cells and cancer: an overview. Stem Cell Rev 3: 249–255. 17955391 10.1007/s12015-007-9002-0

[14] Marhaba R, Zoller M (2004) CD44 in cancer progression: adhesion, migration and growth regulation. J Mol Histol 35: 211–231. 15339042 10.1023/b:hijo.0000032354.94213.69

[15] Hill A, McFarlane S, Johnston PG, Waugh DJ (2006) The emerging role of CD44 in regulating skeletal micrometastasis. Cancer Lett 237: 1–9. 15979783 10.1016/j.canlet.2005.05.006

[16] Jiang L, Deng J, Zhu X, Zheng J, You Y, et al (2012) CD44 rs13347 C>T polymorphism predicts breast cancer risk and prognosis in Chinese populations. Breast Cancer Res 14: R105. 22788972 10.1186/bcr3225 PMC3680922

[17] Gotte M, Yip GW (2006) Heparanase, hyaluronan, and CD44 in cancers: a breast carcinoma perspective. Cancer Res 66: 10233–10237. 17079438 10.1158/0008-5472.CAN-06-1464

[18] Xin Z, Cheng-yi W (2012) Association of CD44 polymorphisms with chemosensitivity to anthracycline-based chemotherapy in breast cancer. Journal of Jilin University Medicine Edition 38: 110–114.

[19] Zhou J, Nagarkatti PS, Zhong Y, Creek K, Zhang J, et al (2010) Unique SNP in CD44 intron 1 and its role in breast cancer development. Anticancer Res 30: 1263–1272. 20530438 PMC4138972

[20] Edge SB, Compton CC (2010) The American Joint Committee on Cancer: the 7th edition of the AJCC cancer staging manual and the future of TNM. Ann Surg Oncol 17: 1471–1474. 20180029 10.1245/s10434-010-0985-4

[21] Therasse P, Arbuck SG, Eisenhauer EA, Wanders J, Kaplan RS, et al (2000) New guidelines to evaluate the response to treatment in solid tumors. European Organization for Research and Treatment of Cancer, National Cancer Institute of the United States, National Cancer Institute of Canada. J Natl Cancer Inst 92: 205–216. 10655437 10.1093/jnci/92.3.205

[22] Barrett JC, Fry B, Maller J, Daly MJ (2005) Haploview: analysis and visualization of LD and haplotype maps. Bioinformatics 21: 263–265. 15297300 10.1093/bioinformatics/bth457

[23] Miller SA, Dykes DD, Polesky HF (1988) A simple salting out procedure for extracting DNA from human nucleated cells. Nucleic Acids Res 16: 1215. 3344216 10.1093/nar/16.3.1215 PMC334765

[24] Gauderman W (2006) QUANTO 1.1: A computer program for power and sample size calculations for genetic-epidemiology studies.

[25] Yuan HY, Chiou JJ, Tseng WH, Liu CH, Liu CK, et al (2006) FASTSNP: an always up-to-date and extendable service for SNP function analysis and prioritization. Nucleic Acids Res 34: W635–641. 16845089 10.1093/nar/gkl236 PMC1538865

[26] Lee PH, Shatkay H (2008) F-SNP: computationally predicted functional SNPs for disease association studies. Nucleic Acids Res 36: D820–824. 17986460 10.1093/nar/gkm904 PMC2238878

[27] So JY, Lee HJ, Smolarek AK, Paul S, Wang CX, et al (2011) A novel Gemini vitamin D analog represses the expression of a stem cell marker CD44 in breast cancer. Mol Pharmacol 79: 360–367. 21115634 10.1124/mol.110.068403 PMC3061370

[28] Screaton GR, Bell MV, Jackson DG, Cornelis FB, Gerth U, et al (1992) Genomic structure of DNA encoding the lymphocyte homing receptor CD44 reveals at least 12 alternatively spliced exons. Proc Natl Acad Sci U S A 89: 12160–12164. 1465456 10.1073/pnas.89.24.12160 PMC50718

[29] Telen MJ, Udani M, Washington MK, Levesque MC, Lloyd E, et al (1996) A blood group-related polymorphism of CD44 abolishes a hyaluronan-binding consensus sequence without preventing hyaluronan binding. J Biol Chem 271: 7147–7153. 8636151 10.1074/jbc.271.12.7147

[30] Lopez JI, Camenisch TD, Stevens MV, Sands BJ, McDonald J, et al (2005) CD44 attenuates metastatic invasion during breast cancer progression. Cancer Res 65: 6755–6763. 16061657 10.1158/0008-5472.CAN-05-0863

[31] Bourguignon LY (2001) CD44-mediated oncogenic signaling and cytoskeleton activation during mammary tumor progression. J Mammary Gland Biol Neoplasia 6: 287–297. 11547898 10.1023/a:1011371523994

[32] Bourguignon LY, Singleton PA, Zhu H, Diedrich F (2003) Hyaluronan-mediated CD44 interaction with RhoGEF and Rho kinase promotes Grb2-associated binder-1 phosphorylation and phosphatidylinositol 3-kinase signaling leading to cytokine (macrophage-colony stimulating factor) production and breast tumor progression. J Biol Chem 278: 29420–29434. 12748184 10.1074/jbc.M301885200

[33] Vazquez A, Grochola LF, Bond EE, Levine AJ, Taubert H, et al (2010) Chemosensitivity profiles identify polymorphisms in the p53 network genes 14-3-3tau and CD44 that affect sarcoma incidence and survival. Cancer Res 70: 172–180. 19996285 10.1158/0008-5472.CAN-09-2218

[34] Winder T, Ning Y, Yang D, Zhang W, Power DG, et al (2011) Germline polymorphisms in genes involved in the CD44 signaling pathway are associated with clinical outcome in localized gastric adenocarcinoma. Int J Cancer 129: 1096–1104. 21105049 10.1002/ijc.25787 PMC3139396

[35] O'Donnell CJ, Cupples LA, D'Agostino RB, Fox CS, Hoffmann U, et al (2007) Genome-wide association study for subclinical atherosclerosis in major arterial territories in the NHLBI's Framingham Heart Study. BMC Med Genet 8 Suppl 1S4. 17903303 10.1186/1471-2350-8-S1-S4 PMC1995605

[36] Zhou J, Nagarkatti PS, Zhong Y, Zhang J, Nagarkatti M (2011) Implications of single nucleotide polymorphisms in CD44 exon 2 for risk of breast cancer. Eur J Cancer Prev 20: 396–402. 21804359 10.1097/CEJ.0b013e3283463943 PMC3968800

[37] Bankfalvi A, Terpe HJ, Breukelmann D, Bier B, Rempe D, et al (1998) Gains and losses of CD44 expression during breast carcinogenesis and tumour progression. Histopathology 33: 107–116. 9762542 10.1046/j.1365-2559.1998.00472.x

[38] He-rui Y, ZHI-hua L, Yong-hui L, Feng-xi S (2009) Research of the relationship between CD44 and resistance of breast cancer chemotherapy Chinese Archives of General Surgery 04.

