# Supplementary figures and images for: CD44 Gene Polymorphisms in Breast Cancer Risk and Prognosis: A Study in North Indian Population
Source: PLoS One. 2013 Aug 5;8(8):e71073. doi: 10.1371/journal.pone.0071073 (PMC3733640; doi:10.1371/journal.pone.0071073)

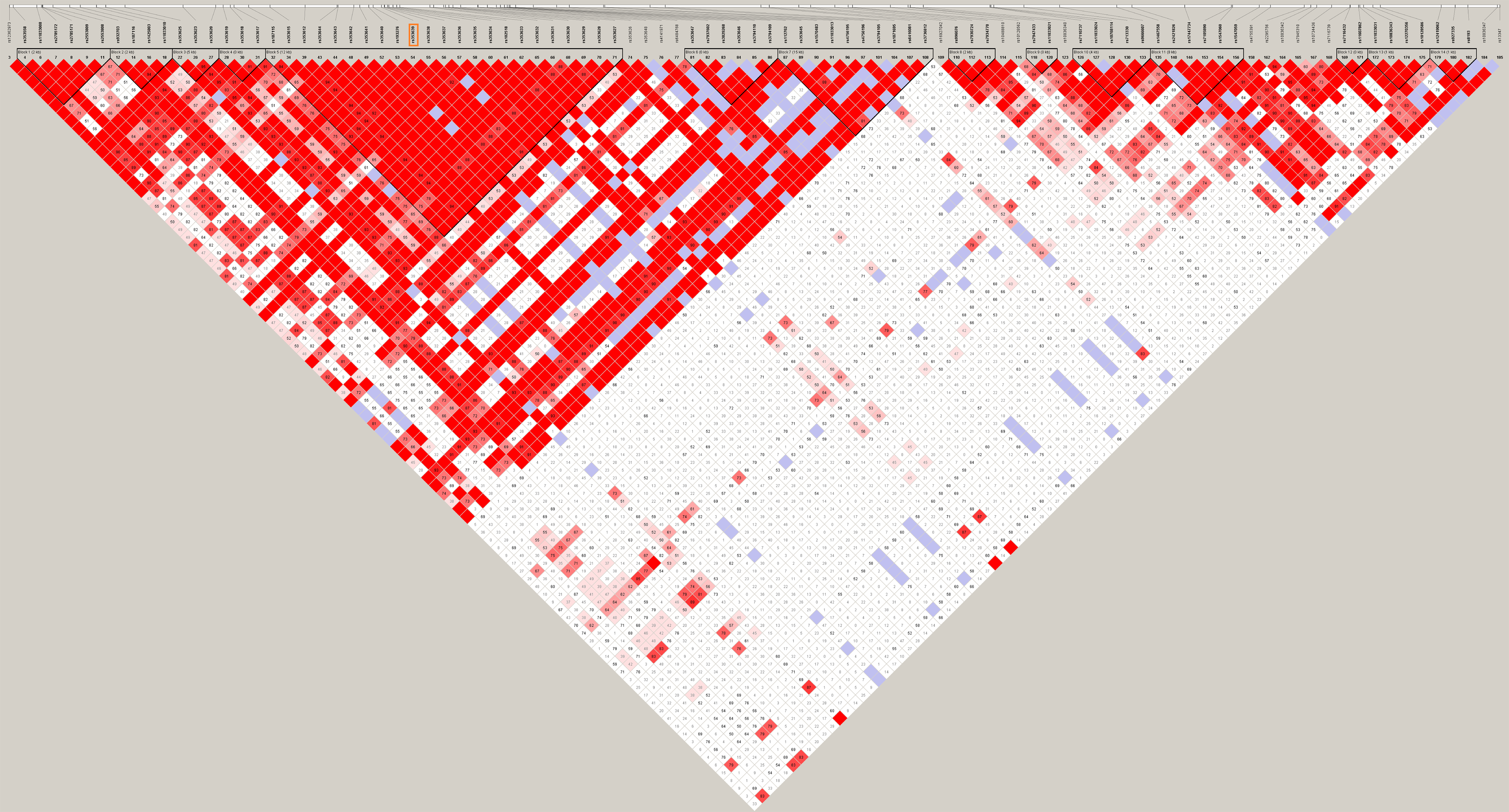

Supplement: Figure S1 — Linkage disequilibrium (LD) plot of CD44 gene in Hapmap- CEU population. (TIF) [file pone.0071073.s001.tif]
